# Elevated Preoperative Systemic Immune-Inflammation Index Independently Predicts 30-Day Mortality After Living Donor Liver Transplantation

**DOI:** 10.3390/life16081373

**Published:** 2026-08-20

**Authors:** Jaesik Park, Jiyoon Bhan, Do Gyeong Lee, Jemin Ko, Minju Kim, Sang Hyun Hong, Chul Soo Park, Hyun Sik Chung

**Affiliations:** Department of Anesthesiology and Pain Medicine, Seoul St. Mary’s Hospital, College of Medicine, The Catholic University of Korea, Seoul 06591, Republic of Korea; jaesik.park@gmail.com (J.P.); jiyun970711@naver.com (J.B.); drdgcatholic@gmail.com (D.G.L.); jaemingoh27@gmail.com (J.K.); minju1025@catholic.ac.kr (M.K.); lifesucks@catholic.ac.kr (S.H.H.); p6c8s17@catholic.ac.kr (C.S.P.)

**Keywords:** systemic immune-inflammation index, living donor liver transplantation, end-stage liver disease, model for end-stage liver disease, mortality, neutrophil-to-lymphocyte ratio, biomarker, prognosis

## Abstract

**Background:** Systemic inflammation significantly impacts graft survival and clinical outcomes following living donor liver transplantation (LDLT). The systemic immune-inflammation index (SII), which integrates peripheral neutrophil, platelet, and lymphocyte counts, has shown prognostic value in various clinical settings, but its role in LDLT has not been thoroughly investigated. **Methods:** This retrospective cohort study included 378 consecutive adult patients with end-stage liver disease (ESLD) who underwent primary LDLT between March 2016 and February 2025. The SII was calculated as (neutrophil × platelet)/lymphocyte. Spearman correlation analysis was used to assess the relationships between the preoperative SII, neutrophil-to-lymphocyte ratio (NLR), and platelet-to-lymphocyte ratio (PLR) and clinical outcomes, including the preoperative Model for End-Stage Liver Disease (MELD) score, duration of mechanical ventilation, and lengths of stay in the intensive care unit (ICU) and hospital. Receiver operating characteristic (ROC) curve analysis determined the optimal cut-off values for predicting 30-day mortality, and the areas under the curve (AUROCs) were compared using the DeLong test. Multivariable logistic regression was used to identify independent predictors of 30-day mortality. **Results:** Among 378 analyzable patients, the 30-day mortality rate was 7.9% (30/378). Both the SII and the NLR were significantly higher in non-survivors than in survivors (SII: median 368.8 vs. 169.1, *p* < 0.001; NLR: 6.0 vs. 2.4, *p* < 0.001), whereas the PLR did not differ significantly. On ROC analysis for 30-day mortality, the NLR and the SII showed comparable discrimination (NLR AUROC = 0.729, 95% CI: 0.63–0.82; SII AUROC = 0.705, 95% CI: 0.60–0.80; DeLong *p* = 0.43), both exceeding the PLR (AUROC = 0.541). The optimal SII cut-off was 275 × 10^9^ cells/L (sensitivity 70.0%; specificity 69.5%). Patients with an SII ≥ 275 × 10^9^ cells/L had significantly lower 30-day survival than those below the cut-off (83.5% vs. 96.4%; log-rank *p* < 0.001). On multivariable logistic regression adjusting for age and MELD score as continuous variables, an SII ≥ 275 × 10^9^ cells/L remained an independent predictor of 30-day mortality (aOR = 3.78; 95% CI: 1.60–8.93; *p* = 0.002); the MELD score was also independently predictive (aOR = 1.04 per point; 95% CI: 1.01–1.08; *p* = 0.018). The association persisted when the SII was modelled continuously (aOR = 1.60 per unit log SII; 95% CI: 1.08–2.38; *p* = 0.020). At the 275 cut-off, sensitivity for 30-day death was 70.0% and the positive predictive value 16.5%. **Conclusions:** The preoperative SII and NLR are simple, inexpensive, CBC-derived inflammatory indices significantly associated with 30-day mortality after LDLT for ESLD. An elevated preoperative SII independently predicts early post-transplant mortality and may aid perioperative risk stratification, although it does not outperform the NLR.

## 1. Introduction

End-stage liver disease (ESLD), which includes decompensated liver cirrhosis, acute-on-chronic liver failure (ACLF), and hepatocellular carcinoma (HCC), is a significant global health concern, causing over one million deaths annually worldwide [1,2]. Liver transplantation (LT) is the only definitive cure for ESLD. Living donor liver transplantation (LDLT) has become an essential alternative due to the ongoing scarcity of deceased donor organs, especially in Asia, the Middle East, and Eastern Europe [3,4].

Despite considerable advancements in surgical techniques, immunosuppressive treatments, and perioperative critical care, the 30-day post-transplant mortality rate at major LDLT centers remains between 5% and 12% [5,6]. The post-transplant recovery is affected by a combination of factors, such as the severity of pre-existing liver disease, surgical stress, ischemia–reperfusion injury, and the recipient’s immune response [7,8,9]. Therefore, accurate preoperative identification of high-risk recipients is crucial. This not only aids in patient selection and donor evaluation, but also helps in guiding informed consent, optimizing resource allocation, and implementing specific perioperative interventions.

The Model for End-Stage Liver Disease (MELD) score, which includes serum bilirubin, international normalized ratio (INR), and creatinine, is the current standard for organ allocation and waitlist prioritization [10]. However, MELD’s ability to predict post-LDLT mortality is limited. This is because it primarily assesses hepatic synthetic dysfunction and renal impairment, failing to account for systemic inflammatory burden, immune status, or physiological reserve—all of which are critical to post-transplant outcomes [11,12].

Systemic inflammation is closely linked to both ESLD progression and post-transplant complications. Patients with cirrhosis often experience a well-documented state of both immune hyperactivation and immune paresis. This condition, known as cirrhosis-associated immune dysfunction (CAID), is caused by chronic gut bacterial translocation, lipopolysaccharide (LPS)-mediated Kupffer cell activation, and progressive lymphocyte depletion [13,14]. This immune-inflammatory imbalance makes recipients more vulnerable to post-transplant infections, primary non-function, acute cellular rejection (ACR), and multi-organ dysfunction syndrome (MODS) [15,16].

Peripheral blood hematologic indices, derived from routine complete blood counts (CBCs), have garnered significant interest as accessible, inexpensive, and reproducible indicators of systemic inflammation [17]. Among these, the neutrophil-to-lymphocyte ratio (NLR), platelet-to-lymphocyte ratio (PLR), and monocyte-to-lymphocyte ratio (MLR) have been explored as prognostic markers in various surgical and oncological contexts [18,19]. However, these single-ratio indices may not fully capture the complex inflammatory environment characteristic of ESLD.

The systemic immune-inflammation index (SII), calculated as (neutrophil count × platelet count)/lymphocyte count, was first introduced by Hu et al. (2014) in the context of hepatocellular carcinoma [20]. By integrating three distinct hematologic compartments—innate immune hyperactivation (neutrophilia), adaptive immune depletion (lymphopenia), and hemostatic-inflammatory platelet activity—SII provides a more comprehensive reflection of systemic immune-inflammatory status than NLR, PLR, or MLR alone [20,21]. SII has since demonstrated prognostic value across solid tumors, cardiac surgery, sepsis, and COVID-19 [22,23,24,25]. Despite this, its specific prognostic utility in the context of LDLT for ESLD has not yet been systematically assessed.

This study aimed to: (1) evaluate the correlations between the preoperative SII and key perioperative outcomes following LDLT, including the MELD score, 30-day mortality, duration of mechanical ventilation, and ICU and hospital length of stay; (2) determine the optimal SII cut-off for predicting 30-day mortality using ROC analysis; (3) compare the prognostic performance of the SII with that of the NLR and PLR; and (4) identify independent predictors of 30-day mortality using multivariable logistic regression.

## 2. Materials and Methods

### 2.1. Study Design and Ethical Approval

This single-center retrospective cohort study was conducted at Seoul St. Mary’s Hospital, The Catholic University of Korea, a tertiary academic liver transplantation center. The study protocol was approved by the Institutional Review Board (IRB Approval No.: KC26RISI0192) and adhered to the ethical principles of the Declaration of Helsinki. Individual informed consent was waived due to the retrospective nature of the study and the use of de-identified data. The study also received approval by the Data Review Board (DRB) of Seoul St. Mary’s Hospital, a board established through the Medical Data-Driven Hospital Support Project of the Korea Health Information Service (KHIS), which is funded by the Ministry of Health and Welfare, Republic of Korea.

### 2.2. Study Population

A total of 412 patients who underwent LDLT at our institution between March 2016 and February 2025 were initially screened. After applying exclusion criteria, 378 patients were included in the final analysis. Exclusion criteria were: (1) multi-organ transplantation (*n* = 9); (2) active infection within 30 days prior to transplantation that could confound inflammatory markers (*n* = 8); (3) hematologic malignancy (leukemia, lymphoma, multiple myeloma) or history of splenectomy that could affect CBC parameters (*n* = 10); and (4) loss to follow-up before 30 days post-transplantation (*n* = 7). Inclusion criteria were: (1) age ≥ 19 years; (2) diagnosis of ESLD requiring LDLT; (3) availability of preoperative CBC data within 24 h prior to transplantation; and (4) availability of complete perioperative clinical data and follow-up through at least 30 days post-transplantation (or until death, for patients who died within 30 days).

### 2.3. Data Collection

Data were extracted from the electronic medical records (EMR) system of Seoul St. Mary’s Hospital. Demographic variables, clinical characteristics (etiology of ESLD, presence of HCC, and comorbidities), and donor/surgical variables were collected. Preoperative laboratory data obtained within 24 h prior to LDLT included complete blood count (CBC), liver function tests, renal function indices, coagulation profiles, and inflammatory markers (C-reactive protein, [CRP]). The primary outcome was 30-day all-cause mortality. Secondary outcomes included mechanical ventilation duration (hours), ICU stay (days), total hospital stay (days), and postoperative complications (infectious complications, early allograft dysfunction [EAD], acute rejection, acute kidney injury requiring renal replacement therapy, and reoperation within 30 days). Laboratory values are reported in the units used by the institutional laboratory (CRP in mg/dL). Transfusion was recorded as the total volume of packed red blood cells administered over the entire perioperative period. Acute rejection was not systematically coded in the institutional database during the study period and was therefore not analysed; the secondary outcomes reported are infection, early allograft dysfunction, reoperation, intraoperative continuous renal replacement therapy, and preoperative haemodialysis.

### 2.4. Immunosuppression Protocol

All patients received a standardized triple immunosuppression regimen consisting of tacrolimus, mycophenolate mofetil (MMF), and corticosteroids. Tacrolimus dosing was adjusted to maintain a target trough blood level of 7–10 ng/mL during the first postoperative month, with subsequent titration based on clinical response and renal function. Corticosteroids were tapered and withdrawn one month after surgery. MMF was continued for six months post-transplantation and then discontinued if no rejection episodes occurred. Splenectomy and local graft infusion were not performed in this cohort. This uniform immunosuppression protocol minimizes pharmacological confounding when interpreting the SII as a preoperative prognostic biomarker, as all SII measurements were obtained preoperatively, prior to any immunosuppressive exposure.

### 2.5. Calculation of Inflammatory Indices

The following inflammatory indices were calculated from preoperative CBC parameters:

(1) SII = (Platelet count [×10^9^/L] × Neutrophil count [×10^9^/L])/Lymphocyte count [×10^9^/L];
(2) NLR = Neutrophil count [×10^9^/L]/Lymphocyte count [×10^9^/L];
(3) PLR = Platelet count [×10^9^/L]/Lymphocyte count [×10^9^/L].

### 2.6. Statistical Analysis

The final analytic sample comprised 378 patients. With 30 thirty-day deaths and three covariates in the multivariable model, the analysis met the conventional events-per-variable threshold of 10. Given the observed 30-day mortality of 7.9%, the number of events constrained the multivariable model to a limited set of pre-specified predictors, and the 30-day estimates should be regarded as exploratory.

Statistical analyses were conducted using IBM SPSS Statistics version 19.0 (IBM Corp., Armonk, NY, USA) and R version 4.3.1 (R Foundation for Statistical Computing, Vienna, Austria) utilizing the ‘pROC’ and ‘survival’ packages. The Shapiro–Wilk test assessed the normality of continuous variables. Normally distributed variables were expressed as mean ± standard deviation (SD) and compared using Student’s *t*-test, while non-normally distributed variables were presented as median (interquartile range, IQR) and compared using the Mann–Whitney *U* test. Categorical variables were compared using the chi-square or Fisher’s exact test. Spearman’s rank correlation coefficients were used to assess relationships between inflammatory indices and continuous outcomes. ROC curves were constructed for 30-day mortality, with optimal cut-off values determined by the Youden index and AUCs compared using the DeLong test. Multivariable logistic regression models identified independent predictors, with model fit assessed by the Hosmer-Lemeshow test. Kaplan–Meier survival analysis with the log-rank test was compared across SII groups. The primary endpoint for all analyses was 30-day mortality, the period most directly attributable to the perioperative inflammatory state. A two-tailed *p*-value < 0.05 was considered statistically significant.

## 3. Results

### 3.1. Baseline Characteristics

A total of 378 patients (mean age 51.8 ± 9.1 years; 70.1% male) were analyzed. Hepatocellular carcinoma (HCC) was present in 43.4% of patients, and the mean preoperative MELD score was 17.1 ± 10.6. The median preoperative SII was 179.9 × 10^9^/L (IQR: 98.1–372.6). The 30-day all-cause mortality rate was 7.9% (30/378). Baseline characteristics, stratified by 30-day survival status, are summarized in Table 1. In summary, non-survivors had significantly higher preoperative SII and NLR values, higher MELD scores, higher CRP levels, and greater intraoperative packed red blood cell (pRBC) requirements than survivors, whereas the PLR did not differ significantly between groups.

### 3.2. Correlation Analysis

Spearman correlation analysis showed that both the SII and the NLR were significantly correlated with the preoperative MELD score, duration of mechanical ventilation, and 30-day mortality, whereas correlations with ICU and hospital length of stay were weak and non-significant. The NLR generally showed numerically stronger correlations than the SII (e.g., with the MELD score, r = 0.50 vs. 0.32; with mechanical ventilation duration, r = 0.39 vs. 0.23), while the PLR was only weakly correlated with the outcomes examined. All significant correlation coefficients were weak-to-moderate in magnitude, indicating that these indices describe associative strength rather than establishing the predictive superiority of any single index. Full correlation data are presented in Table 2.

### 3.3. ROC Curve Analysis and Optimal Cutoff Values

ROC curve analysis was performed for 30-day mortality. The NLR and the SII showed comparable discrimination (NLR AUROC = 0.729, 95% CI: 0.63–0.82; SII AUROC = 0.705, 95% CI: 0.60–0.80), and both exceeded the PLR (AUROC = 0.541, 95% CI: 0.42–0.65). The optimal SII cut-off identified by the Youden index was 275 × 10^9^ cells/L (sensitivity 70.0%; specificity 69.5%). Formal comparison of the two best-performing indices using the DeLong test showed no statistically significant difference between the SII and NLR AUROCs (*p* = 0.43). Thus, in this cohort the SII did not provide discrimination superior to that of the NLR; the two indices performed similarly, and both outperformed the PLR. Full ROC data are presented in Table 3.

### 3.4. Multivariate Logistic Regression Analysis

Covariates for the multivariable model were pre-specified on clinical grounds rather than selected by statistical screening: age and the MELD score were retained because they are the conventional determinants of early post-transplant mortality, and the number of covariates was limited to three by the events-per-variable constraint (30 deaths; events per variable = 10). Age and MELD were modelled as continuous variables to avoid the loss of information caused by dichotomisation. Univariable associations for all candidate variables are reported in Supplementary Table S1. Multivariable logistic regression confirmed that an SII ≥ 275 × 10^9^ cells/L was an independent predictor of 30-day mortality. After adjustment for age and MELD score as continuous variables, an SII ≥ 275 × 10^9^ cells/L was associated with an adjusted odds ratio (aOR) of 3.78 (95% CI: 1.60–8.93; *p* = 0.002). The MELD score was also an independent predictor (aOR = 1.04 per point; 95% CI: 1.01–1.08; *p* = 0.018), whereas age was not (aOR = 1.00 per year; 95% CI: 0.96–1.04; *p* = 0.90). When the SII was modelled continuously, each unit increase in log SII was associated with an aOR of 1.60 (95% CI: 1.08–2.38; *p* = 0.020), indicating that the association does not depend on dichotomisation. In a model additionally adjusted for CRP, the SII remained independently predictive (aOR = 3.47; 95% CI: 1.46–8.24; *p* = 0.005). The full multivariable results are presented in Table 4.

### 3.5. Secondary Postoperative Outcomes

Secondary postoperative outcomes are summarised in Table 5. A high preoperative SII was significantly associated with infection, early allograft dysfunction, and the need for intraoperative continuous renal replacement therapy, but not with reoperation or preoperative haemodialysis. Acute rejection was not systematically coded in the institutional database during the study period and is therefore not reported.

### 3.6. Sensitivity Analyses and Internal Validation

Because the SII cut-off was derived by maximising the Youden index in the same sample in which it was evaluated, we performed additional analyses to assess the robustness of the findings. First, the adjusted model was repeated across a range of cut-off values (Table 6). The association between an elevated SII and 30-day mortality was significant across the central range and attenuated at the extremes, where group sizes became unbalanced. Second, modelling the SII as a continuous variable, which requires no cut-off, gave an aOR of 1.60 per unit log SII (95% CI: 1.08–2.38; *p* = 0.020).

Third, bootstrap internal validation with 1000 resamples gave an apparent area under the receiver operating characteristic curve (AUROC) of 0.705 and an estimated optimism of −0.001, yielding an optimism-corrected AUROC of 0.706. Re-deriving the Youden cut-off within each bootstrap sample produced a median cut-off of 275.5 (interquartile range 275.5–304.4); applying these re-derived cut-offs to the original sample gave a mean sensitivity of 69.3% and specificity of 67.1%, close to the apparent values. The optimism attributable to threshold selection was therefore small in this dataset, although it cannot be assumed negligible in general.

Diagnostic performance at the 275 cut-off was as follows: sensitivity 70.0% (21 of 30 deaths), specificity 69.5%, positive predictive value 16.5%, and negative predictive value 96.4%. Nine of the 30 deaths (30.0%) occurred below the cut-off.

### 3.7. Survival Analysis

**Figure 1:**
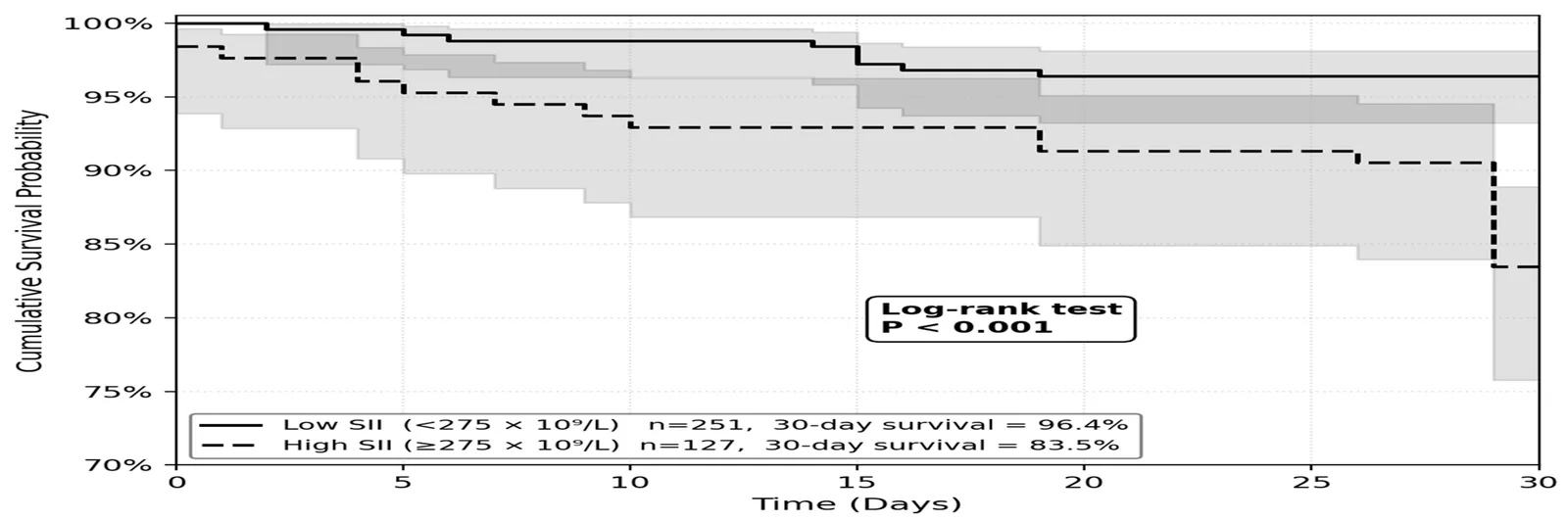
Kaplan–Meier 30-day survival curves stratified by preoperative SII. Patients with a high SII (≥275 × 10^9^ cells/L, dashed line; *n* = 127) had significantly lower 30-day survival (83.5%) than those with a low SII (<275 × 10^9^ cells/L, solid line; *n* = 251; 96.4%). Log-rank *p* < 0.001. Shaded areas = 95% CI. SII: systemic immune-inflammation index.

Kaplan–Meier analysis demonstrated that patients with a preoperative SII ≥ 275 × 10^9^ cells/L had significantly lower 30-day survival than those with an SII < 275 × 10^9^ cells/L (83.5% vs. 96.4%; log-rank *p* < 0.001; Figure 1). This corresponds to a 12.9 percentage-point absolute difference in early post-transplant survival between the two groups (21 deaths among 127 high-SII patients vs. 9 deaths among 251 low-SII patients). Consistent with the multivariable analysis, this finding indicates that an elevated preoperative SII identifies recipients at substantially higher risk of early post-transplant death.

## 4. Discussion

### 4.1. Principal Findings

This retrospective cohort study of 378 LDLT recipients demonstrates that the preoperative SII is significantly associated with 30-day mortality, the preoperative MELD score, and the duration of mechanical ventilation, and that an elevated SII (≥275 × 10^9^ cells/L) independently predicts 30-day mortality after adjustment for age and MELD score (aOR = 3.78). When compared directly with other CBC-derived indices, the SII performed similarly to the NLR—which showed comparable or numerically stronger associations and discrimination—and both outperformed the PLR. Rather than establishing the superiority of any single index, our findings indicate that CBC-based inflammatory indices, particularly the SII and the NLR, provide clinically useful, inexpensive prognostic information for early post-transplant mortality in the LDLT setting.

### 4.2. Biological Rationale for SII in ESLD and LDLT

The biological rationale underpinning SII’s prognostic utility in ESLD is well-grounded. Patients with advanced cirrhosis exhibit systemic immune-inflammatory activation driven by the chronic translocation of bacterial products across a highly permeable, dysbiotic gut epithelium [26,27]. This LPS-mediated activation of Kupffer cells and monocytes results in the sustained elaboration of pro-inflammatory cytokines (TNF-alpha, IL-1beta, IL-6), neutrophilic recruitment, and oxidative endothelial injury [13]. Concurrently, CAID leads to profound lymphocyte functional impairment [28]. Furthermore, platelets in ESLD serve dual immunomodulatory roles, releasing pro-inflammatory mediators such as platelet factor 4 (PF4), RANTES (CCL5), and CD40 ligand (CD40L). These mediators promote neutrophil extracellular trap (NET) formation and amplify the systemic inflammatory cascade [29,30]. By incorporating the product of neutrophil and platelet counts divided by lymphocyte count, SII amplifies the prognostic signal when both systemic inflammation and immune paresis are concurrently present—a state particularly characteristic of high-risk ESLD.

### 4.3. Immunosuppression Protocol and Relationship to SII

An important consideration when interpreting SII as a prognostic biomarker is the potential interaction between systemic immune-inflammatory status and the post-transplantation immunosuppression regimen. In this study, all patients received a standardized triple immunosuppression protocol (tacrolimus 7–10 ng/mL trough, MMF, and corticosteroids), which minimized immunosuppression-related heterogeneity as a confounding variable. Critically, SII was measured preoperatively, prior to any immunosuppressive exposure. This preoperative measurement window isolates the patient’s intrinsic immune-inflammatory phenotype—reflective of ESLD-driven systemic inflammatory burden—from any iatrogenic immunomodulation. Tacrolimus-induced lymphocyte suppression and corticosteroid-induced neutrophilia are well-recognized hematologic effects [31,32] that would confound postoperative SII measurement, further justifying the preoperative assessment strategy employed here. The standardized immunosuppression protocol and preoperative timing of SII measurement constitute important methodological safeguards that strengthen the validity of our findings.

### 4.4. Comparison with NLR and PLR

Our data indicate that the SII and the NLR provide comparable prognostic information for 30-day mortality after LDLT, with both outperforming the PLR. In our cohort, the NLR showed correlations and discrimination at least as strong as those of the SII, and the DeLong test found no significant difference between their AUROCs (*p* = 0.43); we therefore do not claim that the SII is superior to the NLR. Nonetheless, the SII remained a strong independent predictor of 30-day mortality on multivariable analysis, supporting its value as one of several useful CBC-derived markers. This is consistent with evidence from the surgical oncology literature. For instance, in patients with hepatocellular carcinoma undergoing curative resection, the SII demonstrated superior prognostic performance compared to NLR and PLR [20]. Similarly, in patients with acute-on-chronic liver failure, an elevated SII was associated with increased short-term mortality, with an optimal cut-off of approximately 425 × 10^9^ cells/L [33]. Our study extends these findings by demonstrating the prognostic utility of the SII for 30-day mortality, alongside the NLR, within a dedicated LDLT cohort [34]. In the revised analysis, in which age and the MELD score were modelled as continuous variables, the MELD score was itself an independent predictor of 30-day mortality (aOR = 1.04 per point; *p* = 0.018). The SII therefore adds prognostic information to the MELD score rather than substituting for it, and our earlier suggestion that the SII might be especially useful because the MELD score lacked independent predictive value is not supported by the revised analysis and has been withdrawn.

### 4.5. Clinical Interpretation of SII Cutoff Values

The identified SII cutoff of 275 × 10^9^ cells/L for predicting 30-day post-LDLT mortality is lower than cutoffs reported in acute-on-chronic liver failure (425 × 10^9^/L) [33] and HCC resection cohorts (330 × 10^9^/L) [20]. This difference may reflect the distinct immunological profile of LDLT recipients, who often present with constitutional thrombocytopenia due to portal hypertension and hypersplenism. This same feature may explain why the SII did not outperform the simpler NLR in our cohort. In ESLD, the platelet component of the SII is governed less by inflammation than by portal hypertension, hypersplenic sequestration, and reduced hepatic thrombopoietin production; consequently, the platelet count is frequently depressed even when systemic inflammation is high, which can blunt the SII and attenuate the incremental information contributed by its platelet term. In this relatively low-MELD ESLD population, the two-component NLR—which is unaffected by platelet sequestration—therefore captured the neutrophil–lymphocyte inflammatory axis at least as effectively as the three-component SII. Rather than a limitation, this observation offers a mechanistic rationale for interpreting CBC-derived indices in ESLD and supports reporting the SII alongside, rather than in place of, the NLR. For 30-day mortality, the SII and the NLR provided comparable discrimination (AUROC 0.705 and 0.729, respectively), consistent with the impact of the preoperative systemic inflammatory burden on early post-transplant outcomes.

### 4.6. Clinical Implications

Our findings have several important clinical implications. First, preoperative SII assessment is simple, inexpensive, and readily available from routine CBC measurements, making it an attractive tool for risk stratification in LDLT candidates. Second, SII’s independent predictive value, beyond traditional prognostic factors such as MELD score, suggests it captures a distinct aspect of the patient’s physiological status. Incorporating SII into existing prognostic algorithms may therefore improve predictive accuracy. Third, although the preoperative SII was significantly associated with the duration of mechanical ventilation, it was not significantly correlated with ICU or hospital length of stay in this cohort; the prognostic value of the SII therefore appears to relate primarily to mortality and early ventilatory course rather than to overall resource utilization. Preoperative interventions targeting inflammatory burden, such as nutritional optimization, treatment of subclinical infections, or rifaximin-based gut decontamination—represent testable strategies for improving outcomes in high-risk patients, though prospective evaluation is required.

### 4.7. Strengths and Limitations

This study has several strengths. First, we included a relatively large and homogeneous cohort from a single high-volume center, utilizing standardized immunosuppression and surgical protocols. Second, we evaluated the SII for the 30-day mortality endpoint using complementary correlation, ROC, Kaplan–Meier, and multivariable analyses, and we benchmarked it directly against the NLR and PLR. Third, we directly compared the SII against two established CBC-derived inflammatory indices, the NLR and the PLR, using formal ROC and DeLong testing. Fourth, measuring SII preoperatively eliminates confounding factors related to post-transplant immunosuppressive therapy. However, several limitations should be acknowledged. This was a retrospective, single-center study, which limits the generalizability of our findings. The study population, predominantly Asian patients with HBV-related cirrhosis, may further limit applicability to Western LDLT cohorts. With 30 observed 30-day events and three covariates, the multivariable model had an events-per-variable ratio of 10, which meets the conventionally recommended minimum. Nonetheless, given the modest absolute number of events, the estimates should still be interpreted with caution, and the confidence intervals are correspondingly wide. Furthermore, SII was measured at a single preoperative time point, without assessing dynamic postoperative changes. Finally, the determined cut-off values require external prospective validation before clinical adoption. A further limitation is that the SII cut-off of 275 × 10^9^ cells/L was identified by maximising the Youden index in the same cohort in which it was subsequently evaluated, a procedure that is optimistically biased. We addressed this by repeating the analysis across a range of cut-offs, by modelling the SII continuously, and by bootstrap internal validation, all of which supported the robustness of the association; nevertheless the value of 275 should be regarded as a data-derived estimate specific to this cohort rather than a validated clinical threshold. In addition, the diagnostic performance of that threshold is modest: sensitivity was 70.0% and the positive predictive value 16.5%, so nearly one third of deaths occurred below the cut-off and most patients above it survived. Finally, no decision-curve analysis or external validation was undertaken, and both would be required before the SII could be considered for clinical decision-making.

### 4.8. Future Directions

Future multicenter studies are needed to validate SII cutoff values in LDLT recipients. Dynamic postoperative SII kinetics warrant investigation as predictors of graft function and late outcomes. Developing composite scoring tools that incorporate SII, MELD score, donor age, graft-to-recipient weight ratio, and CRP may offer superior prognostic accuracy. Machine learning-based models that incorporate SII alongside multiparametric clinical and donor data represent a promising avenue for personalized risk prediction.

This retrospective cohort study shows that an elevated preoperative SII is independently associated with 30-day mortality after LDLT for ESLD. After adjustment for age and MELD score as continuous variables, an SII ≥ 275 × 10^9^ cells/L carried an adjusted odds ratio of 3.78 (95% CI: 1.60–8.93), and the association persisted when the SII was modelled continuously. The MELD score remained an independent predictor in the same model, so the SII should be regarded as providing information additional to, not in place of, established risk indices. Its discrimination was comparable to that of the NLR and superior to that of the PLR, and an elevated SII was also associated with infection, early allograft dysfunction, and the need for intraoperative renal replacement therapy.

These findings should be interpreted with caution. At the 275 cut-off, the sensitivity was 70.0% and the positive predictive value 16.5%, meaning that most patients above the cut-off survived and that 30% of deaths occurred below it. The cut-off was derived and evaluated in the same cohort, and no decision-curve analysis or external validation was performed. The SII is therefore not adequate as a stand-alone triage instrument, and these data do not support using it to modify postoperative surveillance. What the results support is narrower: the SII is an inexpensive, routinely available marker that may contribute to perioperative risk assessment alongside established indices, and that merits evaluation in prospective, externally validated studies.

## Figures and Tables

**Table 1:** Baseline Characteristics of the Study Population (*N* = 378), Stratified by 30-Day Survival.

Characteristic	All (*n* = 378)	Survivors (*n* = 348)	Non-Survivors (*n* = 30)	*p*-Value
Age, years, mean ± SD	51.8 ± 9.1	52.0 ± 8.9	50.1 ± 11.8	0.391
Male sex, *n* (%)	265 (70.1)	244 (70.1)	21 (70.0)	1.000
BMI, kg/m^2^, mean ± SD	24.6 ± 3.7	24.6 ± 3.7	24.4 ± 4.4	0.850
MELD score, median (IQR)	14.0 (9.0–24.0)	13.0 (8.0–23.0)	22.5 (15.0–35.0)	0.001
HCC present, *n* (%)	164 (43.4)	156 (44.8)	8 (26.7)	0.057
SII, ×10^9^/L, median (IQR)	179.9 (98.1–372.6)	169.1 (95.4–338.8)	368.8 (191.3–759.4)	<0.001
NLR, median (IQR)	2.6 (1.5–5.2)	2.4 (1.5–4.5)	6.0 (3.0–10.4)	<0.001
PLR, median (IQR)	74.1 (53.6–110.1)	73.9 (53.4–109.3)	78.4 (58.8–119.3)	0.459
Albumin, g/dL, median (IQR)	3.0 (2.7–3.5)	3.0 (2.7–3.5)	3.0 (2.6–3.3)	0.606
CRP, mg/dL, median (IQR)	0.5 (0.1–1.4)	0.5 (0.1–1.2)	1.8 (0.7–3.4)	<0.001
pRBC, units, median (IQR)	9.0 (4.0–14.0)	8.0 (4.0–13.0)	15.0 (8.0–21.0)	0.002
GRWR, %, mean ± SD	1.2 ± 0.4	1.2 ± 0.4	1.2 ± 0.6	0.669

BMI: body mass index; HCC: hepatocellular carcinoma; SII: systemic immune-inflammation index; NLR: neutrophil-to-lymphocyte ratio; PLR: platelet-to-lymphocyte ratio; CRP: C-reactive protein; pRBC: packed red blood cells; GRWR: graft-to-recipient weight ratio; IQR: interquartile range; SD: standard deviation.

**Table 2:** Correlation Between Inflammatory Indices and Clinical Parameters.

Clinical Parameter	SII	NLR	PLR
Preoperative MELD	r = 0.32 **	r = 0.50 **	r = −0.12 *
Mechanical ventilation time	r = 0.23 **	r = 0.39 **	r = −0.01
ICU stay	r = 0.08	r = 0.05	r = 0.06
Hospital stay	r = 0.07	r = 0.07	r = 0.00
30-day mortality	r = 0.19 **	r = 0.21 **	r = 0.04

* *p* < 0.05; ** *p* < 0.01. SII: systemic immune-inflammation index; NLR: neutrophil-to-lymphocyte ratio; PLR: platelet-to-lymphocyte ratio; MELD: model for end-stage liver disease; ICU: intensive care unit.

**Table 3:** ROC Curve Analysis for Prediction of Mortality.

Index	AUROC (95% CI)	Cut-Off	Sensitivity (%)	Specificity (%)
SII	0.705 (0.60–0.80)	275 × 10^9^/L	70.0	69.5
NLR	0.729 (0.63–0.82)	2.6	86.7	53.4
PLR	0.541 (0.42–0.65)	69.5	70.0	43.4

SII: systemic immune-inflammation index (×10^9^ cells/L); NLR: neutrophil-to-lymphocyte ratio; PLR: platelet-to-lymphocyte ratio; AUROC: area under the receiver operating characteristic curve; CI: confidence interval.

**Table 4:** Multivariable Logistic Regression Analysis for 30-Day Mortality.

Variable	aOR	95% CI	*p*-Value
SII ≥ 275 × 10^9^ cells/L	3.78	1.60–8.93	0.002
Age (per year)	1.00	0.96–1.04	0.897
MELD score (per point)	1.04	1.01–1.08	0.018

aOR: adjusted odds ratio; CI: confidence interval; SII: systemic immune-inflammation index; MELD: model for end-stage liver disease.

**Table 5:** Secondary postoperative outcomes according to preoperative SII group.

Outcome, % (*n*/*N*)	Low SII (<275)	High SII (≥275)	*p*-Value
Infection	2.0 (5/251)	8.2 (10/122)	0.009
Early allograft dysfunction	11.8 (29/246)	24.2 (29/120)	0.004
Intraoperative continuous renal replacement therapy	2.8 (7/250)	19.0 (24/126)	<0.001
Reoperation	4.5 (11/246)	7.4 (9/121)	0.329
Preoperative haemodialysis	11.6 (29/251)	16.5 (21/127)	0.199

Data are percentages with counts. *p*-values from Fisher’s exact test. SII: systemic immune-inflammation index.

**Table 6:** Sensitivity of the adjusted association to the choice of SII cut-off.

SII Cut-Off (×10^9^ Cells/L)	*n* Above Cut-Off	aOR (95% CI)	*p*-Value
150	221	2.84 (1.03–7.83)	0.043
200	175	2.07 (0.88–4.84)	0.094
250	137	3.30 (1.40–7.77)	0.006
275 (Youden)	127	3.78 (1.60–8.93)	0.002
300	121	3.43 (1.48–7.96)	0.004
350	102	2.39 (1.07–5.34)	0.034
400	84	2.00 (0.88–4.52)	0.096

All models adjusted for age and MELD score as continuous variables. aOR: adjusted odds ratio; CI: confidence interval.

## Data Availability

The data presented in this study are available on request from the corresponding author. The data are not publicly available due to privacy and ethical restrictions related to patient health information.

## Supplementary Materials

The following supporting information can be downloaded at: https://www.mdpi.com/article/10.3390/life16081373/s1, Table S1: Univariable logistic regression for 30-day all-cause mortality after living donor liver transplantation; Table S2: Sensitivity of the adjusted association between the SII and 30-day mortality to the choice of cut-off; Table S3: Bootstrap internal validation.

## Abbreviations

The following abbreviations are used in this manuscript:

SII	Systemic immune-inflammation index
LDLT	Living donor liver transplantation
ESLD	End-stage liver disease
NLR	Neutrophil-to-lymphocyte ratio
PLR	Platelet-to-lymphocyte ratio
MELD	Model for End-Stage Liver Disease
CAID	Cirrhosis-associated immune dysfunction
