# Supplementary material for: Elevated Preoperative Systemic Immune-Inflammation Index Independently Predicts 30-Day Mortality After Living Donor Liver Transplantation
Source: Life (Basel). 2026 Aug 20;16(8):1373. doi: 10.3390/life16081373 (PMC13514752; doi:10.3390/life16081373)
Supplement: Supplementary file 1 [file life-16-01373-s001.zip › life-4435579-supplementary.pdf]

Supplementary Material

Elevated Preoperative Systemic Immune-Inflammation Index Independently Predicts 30-Day Mortality After Living Donor Liver Transplantation

Journal: Life (MDPI) | Manuscript ID: life-4435579

Table S1. Univariable logistic regression for 30-day all-cause mortality after living donor liver transplantation (n = 378; 30 events).

Variable	Unit / comparison	n	Unadjusted OR (95% CI)	P-value
Age	*per year*	378	0.978 (0.941–1.017)	0.270
Male sex	*vs female*	378	0.995 (0.441–2.244)	0.989
Body mass index	*per kg/m²*	378	0.989 (0.893–1.094)	0.824
MELD score	*per point*	378	1.062 (1.030–1.096)	**<0.001**
Hepatocellular carcinoma	*present vs absent*	378	0.448 (0.194–1.033)	**0.060**
SII (log-transformed)	*per unit log*	378	1.945 (1.345–2.812)	**<0.001**
SII ≥ 275 $\times 10^9$/L	*vs < 275*	378	5.327 (2.361–12.017)	**<0.001**
NLR	*per unit*	378	1.075 (1.029–1.123)	**0.001**
PLR	*per unit*	378	1.003 (0.998–1.008)	0.291
C-reactive protein	*per mg/dL*	378	1.285 (1.125–1.468)	**<0.001**
Albumin	*per g/dL*	378	0.839 (0.421–1.670)	0.617
pRBC transfusion	*per unit*	377	1.064 (1.029–1.102)	**<0.001**
Graft-to-recipient weight ratio	*per %*	354	1.395 (0.520–3.739)	0.508

Each row represents a separate univariable logistic regression model with 30-day all-cause mortality as the dependent variable. Odds ratios are per unit shown. P-values in bold are < 0.10. The SII is presented both as a log-transformed continuous variable and dichotomised at the 275 × 10^9 cells/L cut-off; the dichotomised form is shown for reference only, as the cut-off was derived within this cohort. CI, confidence interval; MELD, Model for End-Stage Liver Disease; NLR, neutrophil-to-lymphocyte ratio; OR, odds ratio; PLR, platelet-to-lymphocyte ratio; pRBC, packed red blood cells; SII, systemic immune-inflammation index.

Note on covariate selection. Covariates for the multivariable model were pre-specified on clinical grounds rather than selected by statistical screening. Age and the MELD score were retained because they are the conventional determinants of early post-transplant mortality, and the number of covariates was limited to three by the events-per-variable constraint (30 events). This table is provided for transparency so that readers can see the univariable associations of all candidate variables, including those not entered into the multivariable model. We note in particular that age was not associated with mortality on univariable analysis (P = 0.270) while C-reactive protein was (P < 0.001); a model additionally adjusted for C-reactive protein is reported in the main text, in which the SII remained independently predictive.

Table S2. Sensitivity of the adjusted association between the SII and 30-day mortality to the choice of cut-off.

SII cut-off ($\times 10^9$ cells/L)	n at or above cut-off	Adjusted OR (95% CI)	P-value
150	221	2.84 (1.03–7.83)	0.043
200	175	2.07 (0.88–4.84)	0.094
250	137	3.30 (1.40–7.77)	0.006
**275 (Youden-derived)**	**127**	**3.78 (1.60–8.93)**	**0.002**
300	121	3.43 (1.48–7.96)	0.004
350	102	2.39 (1.07–5.34)	0.034
400	84	2.00 (0.88–4.52)	0.096

All models adjusted for age and MELD score as continuous variables. The association is significant across the central range of cut-offs and attenuates at the extremes, where the groups become markedly unbalanced. The value of 275 × 10^9 cells/L was derived by maximising the Youden index within this cohort and should be regarded as a data-derived estimate rather than a validated clinical threshold. CI, confidence interval; OR, odds ratio; SII, systemic immune-inflammation index.

Table S3. Bootstrap internal validation (1000 resamples).

Quantity	Value
Apparent AUROC (SII, 30-day mortality)	0.705

Estimated optimism	−0.001
Optimism-corrected AUROC	0.706
Median bootstrap-derived Youden cut-off	275.5 (IQR 275.5–304.4)
Mean sensitivity of bootstrap cut-offs applied to the original sample	69.3%
Mean specificity of bootstrap cut-offs applied to the original sample	67.1%

Optimism was estimated by Harrell's bootstrap procedure. The negligible optimism reflects the fact that a single pre-measured continuous marker was evaluated rather than a fitted multivariable prediction rule. AUROC, area under the receiver operating characteristic curve; IQR, interquartile range.
